# d-Cystine di(m)ethyl ester reverses the deleterious effects of morphine on ventilation and arterial blood gas chemistry while promoting antinociception

**DOI:** 10.1038/s41598-021-89455-2

**Published:** 2021-05-11

**Authors:** Benjamin Gaston, Santhosh M. Baby, Walter J. May, Alex P. Young, Alan Grossfield, James N. Bates, James M. Seckler, Christopher G. Wilson, Stephen J. Lewis

**Affiliations:** 1 Herman B Wells Center for Pediatric Research, Indiana University School of Medicine, Indianapolis, IN 46202 USA; 2 Pediatric Respiratory Medicine, University of Virginia School of Medicine, Charlottesville, VA 22908 USA; 3 Department of Biochemistry and Biophysics, University of Rochester Medical Center, Rochester, NY 14642 USA; 4 Department of Anesthesia, University of Iowa Hospitals and Clinics, Iowa City, IA 52242 USA; 5 Department of Biomedical Engineering, Case Western Reserve University, Cleveland, OH 44106 USA; 6 Basic Sciences, Division of Physiology, School of Medicine, Loma Linda University, Loma Linda, CA 92350 USA; 7 Department of Pharmacology, Case Western Reserve University, Cleveland, OH 44106 USA; 8 Division of Pulmonology, Allergy and Immunology, Departments of Pediatrics, School of Medicine, Case Western Reserve University, 10900 Euclid Avenue, Cleveland, OH 44106-4984 USA; 9 Present Address: Translational Sciences Treatment Discovery, Galvani Bioelectronics, Inc., 1250 S Collegeville Rd., Collegeville, PA 1r9426 USA

**Keywords:** Drug discovery, Systems biology

## Abstract

We have identified thiolesters that reverse the negative effects of opioids on breathing without compromising antinociception. Here we report the effects of d-cystine diethyl ester (d-cystine diEE) or d-cystine dimethyl ester (d-cystine diME) on morphine-induced changes in ventilation, arterial-blood gas chemistry, A-a gradient (index of gas-exchange in the lungs) and antinociception in freely moving rats. Injection of morphine (10 mg/kg, IV) elicited negative effects on breathing (e.g., depression of tidal volume, minute ventilation, peak inspiratory flow, and inspiratory drive). Subsequent injection of d-cystine diEE (500 μmol/kg, IV) elicited an immediate and sustained reversal of these effects of morphine. Injection of morphine (10 mg/kg, IV) also elicited pronounced decreases in arterial blood pH, pO_2_ and sO_2_ accompanied by pronounced increases in pCO_2_ (all indicative of a decrease in ventilatory drive) and A-a gradient (mismatch in ventilation-perfusion in the lungs). These effects of morphine were reversed in an immediate and sustained fashion by d-cystine diME (500 μmol/kg, IV). Finally, the duration of morphine (5 and 10 mg/kg, IV) antinociception was augmented by d-cystine diEE. d-cystine diEE and d-cystine diME may be clinically useful agents that can effectively reverse the negative effects of morphine on breathing and gas-exchange in the lungs while promoting antinociception. Our study suggests that the d-cystine thiolesters are able to differentially modulate the intracellular signaling cascades that mediate morphine-induced ventilatory depression as opposed to those that mediate morphine-induced antinociception and sedation.

## Introduction

Opioids are given for pain relief in humans and animals but their ability to depress breathing and gas exchange in the lungs is often problematic to the patient^1–5^. Competitive opioid receptor antagonists such as naloxone and naltrexone can overcome opioid-induced respiratory depression (OIRD) but they also reverse the antinociception and sedation elicited by opioids so that their clinical value is limited in many instances such as in the operating room and in post-operative sites where pain relief and sedation are essential to the patient^1–5^. Despite the urgent need to develop drugs that reverse OIRD by mechanisms other than antagonism of opioid receptors, a recent review^5^ concluded that none of the currently available experimental drugs are adequate for therapeutic use in OIRD for a variety of important reasons including that they only minimally improve arterial blood-gas (ABG) chemistry and that all of the drugs need further study of efficacy and toxicity.

Trivedi and colleagues^6^ provided compelling evidence that morphine induces addiction via causing redox-based changes in global DNA methylation and retrotransposon transcription via morphine-induced inhibition of excitatory amino acid transporter type 3 (EAA3)-mediated cysteine uptake into cells. In addition, Trivedi and Deth^7^ proposed pharmacological strategies including administration of N-acetyl-l-cysteine (l-NAC) which elevates intracellular cysteine levels) to reverse the redox-based epigenetic status of drug addiction. Although there is no evidence that blockade of l-cysteine entry into cells has a role in the expression of OIRD, we reasoned that administration of drugs that bypass the EAA3 may overcome OIRD if this indeed is the major mechanism that needs to be overcome. More specifically, the delivery of l-cysteine or D-cysteine to cells via a process that bypasses the EEA3 may have the ability to overcome OIRD. The class of compounds we have focused upon are the ethyl ester and methyl ester versions of reduced and oxidized thiols (disulfides), which are known to rapidly enter cells including neurons in the brain upon systemic administration^8–22^. Our initial publication^22^ reported that systemic injection of l-cysteine ethyl ester (l-CYSee) reversed the negative effects of morphine on ABG chemistry and Alveolar-arterial (A-a) gradient (index of gas exchange in the lungs) anesthetized rats only when the rats had a tracheotomy. Since l-cysteine itself was inert, we assumed that the biological activity of l-CYSee was due to its intracellular entry and initiation of redox-related processes. It appears that the ability of l-CYSee to reverse the negative effects of morphine on breathing and gas-exchange are overridden by the negative effects on the upper airway including collapse of the tongue over the airway^22^.

Accordingly, we have begun to explore whether D-isomers of cysteine and cystine esters show positive effects against OIRD without the negative effects of the L-isomers, in the hope that (i) the intracellular mechanisms of action of thiolesters are not dependent on stereoselective processes whereas the negative effects of the L-isomers are due to them entering into metabolic pathways that D-isomers cannot. We report here the effects of d-cystine ethyl ester (d-cystine diEE) and d-cystine methyl ester (d-cystine diME) on the actions of morphine in freely moving adult male Sprague–Dawley rats. These L-isomers of these thiolesters rapidly enter cells and exert a variety of metabolic effects^8–16^ but we have not found publications regarding the bioactivity of the D-isomers. We report that systemic injection of d-cystine diEE or d-cystine diME elicits a rapid and sustained reversal of the negative effects of morphine on ventilatory parameters, A-a gradient and ABG chemistry whereas it augmented the analgesic actions of the opioid agonist. This pharmacological profile would be advantageous in many clinical settings involving patients who require opioids for essential pain relief (e.g., those just having undergone surgery) and who cannot be administered opioid receptor antagonists to overcome serious ventilatory depression.

## Methods

### Permissions, rats, surgical procedures and blinding of protocols

All studies were carried out in accordance with the NIH Guide for the Care and Use of Laboratory Animals (NIH Publication No. 80-23) revised in 1996. The protocols were approved by the Animal Care and Use Committees of the University of Virginia, Case Western Reserve University and Loma Linda University. In addition, all studies were carried out in compliance with the ARRIVE (Animal Research: Reporting of In Vivo Experiments) guidelines (http://www.nc3rs.org.uk/page.asp?id=1357). Adult male Sprague–Dawley rats (Harlan, Madison, WI, USA) were implanted with jugular vein catheters under 2% isoflurane anesthesia and some rats received femoral arterial catheters^23^. The rats were allowed at least four days to recover from surgery before use. All arterial catheters were flushed daily with heparin solution (50 units of heparin in phosphate-buffered saline at 0.1 M, pH 7.4). All catheters were flushed with phosphate-buffered saline (0.1 M, pH 7.4) approximately four hours before commencement of the experiments. All studies were performed in a quiet laboratory with relative humidity of 49 ± 2% and room temperature of 21.4 ± 0.2 °C. Please note that in the studies described in the main manuscript below, we examined the effects d-cystine diEE against morphine in the plethysmography studies and the effects of d-cystine diME in the arterial blood gas/A- gradient and antinociception studies. Also, please note that the antinociception and ventilatory studies were done in separate groups of rats so as to not complicate the respiratory measurements. The recording (plethysmography, antinociception) sessions and the arterial blood gas assays were done by a particular investigator who administered the opioid, vehicle or test drug such as d-cystine diME. The syringes containing the vehicle or test drug were made up by another investigator, such that the investigator running the actual experiment was blinded to the treatment protocol. In every case, the data files resulting from a particular study were fist collated and analyzed by another investigator in the group.

### Whole-body plethysmography measurement of ventilatory parameters

Ventilatory parameters were recorded in freely moving rats by whole body plethysmography (PLY3223; Data Sciences International, St. Paul, MN) as described previously^23–27^. The rats were placed in individual chambers and given 60 min to acclimatize to allow true resting ventilatory parameters to be established. Study 1—see Supplemental Table S1: Two groups of rats received a bolus injection of morphine (10 mg/kg, IV) and after 15 min, one group received an injection of vehicle (saline) whereas the other received an injection of d-cystine diEE (500 μmol/kg, IV) and ventilatory parameters were recorded for a further 75 min. Study 2—see Supplemental Table S3: Two groups of rats received a bolus injection of morphine (10 mg/kg, IV) and after 15 min, one group received an injection of vehicle (saline) whereas the other received an injection of d-cystine (500 μmol/kg, IV) and ventilatory parameters were recorded for a further 75 min. Study 3—see Supplemental Table S4: Since Trivedi and Deth^7^ proposed that administration of N-acetyl- l-cysteine (l-NAC) may help reverse the redox-based epigenetic status of opioid addiction, we thought it appropriate to also determine whether the thiolester, l-N-acetylcysteine methyl ester (l-NACme), which is a highly cell penetrable reducing agent^21^, would reverse the negative effects of morphine on breathing. Two groups of rats received a bolus injection of morphine (10 mg/kg, IV) and after 15 min, one group received an injection of vehicle (saline) whereas the other received an injection l-NACme (500 μmol/kg, IV)^21^. The rats received another injection of vehicle or l-NACme (500 μmol/kg, IV) 15 min later and ventilatory parameters were recorded for a further 60 min.

Due to the closeness of the body weights of all of the groups of rats, ventilatory data are shown without any corrections for body weight. The provided software (Fine Pointe, BUXCO) constantly corrected digitized values for changes in chamber temperature and humidity. Pressure changes associated with the respiratory waveforms were then converted to volumes (i.e., TV, PIF and PEF) using the algorithm of Epstein and colleagues^28–30^. Specifically, factoring in chamber temperature and humidity, the cycle analyzers filtered the acquired signals, and BUXCO algorithms (Fine Pointe) generated an array of box flow data that identified a waveform segment as an acceptable breath. From that data vector, the minimum and maximum values were determined. Flows at this point were considered to be “box flow” signals. From this array, the minimum and maximum box flow values were determined and multiplied by a compensation factor provided by the selected algorithm^50,51^, thus producing TV, PIF and PEF values that were used to determine accepted and rejected waveforms, with rejected waveforms remaining below 5% throughout all phases of the protocols except for a transient rise in rejection of breaths to 15–20% for 1–2 min after injection of morphine (data not shown).

### Protocols for blood gas measurements and determination of Arterial-alveolar gradient

The changes in pH, pCO_2_, pO_2_ and sO_2_ elicited by injection of morphine (10 mg/kg, IV) in 3 separate groups of freely moving rats (n = 9 rats per group) followed 15 min later by injection of vehicle (saline; 80.0 ± 0.6 days of age; 342 ± 2 g body weight), d-cystine (500 μmol/kg, IV; 79.7 ± 0.4 days; 340 ± 2 g) or d-cystine diME (500 μmol/kg, IV; 79.3 ± 0.4 days; 338 ± 2 g) were determined as detailed previously (49). Arterial blood samples (100 μL) were taken 15 min before and 15 min after injection of morphine (10 mg/kg, IV). The rats then immediately received an injection of vehicle, d-cystine or d-cystine diME and blood samples were taken 5, 15, 30 and 45 min later. The pH, pCO_2_, pO_2_ and sO_2_ were measured using a Radiometer blood-gas analyzer (ABL800 FLEX). The A-a gradient measures difference between alveolar and arterial blood O_2_ concentrations^23,31,32^. A decrease in PaO_2_, without a change in A-a gradient is normally accompanied by an increase in paCO_2_ (as observed here) if it is caused by hypoventilation. Hypoxia is irreversible if caused by shunt. An increased A-a gradient is caused either by oxygen diffusion limitation (usually not readily reversible) or ventilation-perfusion mismatch^23,31,32^. A-a gradient = PAO_2_ − PaO_2_, where PAO_2_ is the partial pressure of alveolar O_2_ and PaO_2_ is pO_2_ in arterial blood. PAO_2_ = [(FiO_2_ × (P_atm_—P_H2O_)—(PaCO_2_/respiratory quotient)], where FiO_2_ is the fraction of O_2_ in inspired air; P_atm_ is atmospheric pressure; P_H2O_ is the partial pressure of H_2_O in inspired air; PaCO_2_ is pCO_2_ in arterial blood; and respiratory quotient (RQ) is the ratio of CO_2_ eliminated/O_2_ consumed. We took FiO_2_ of room-air to be 21% = 0.21, P_atm_ to be 760 mmHg, and P_H2O_ to be 47 mmHg^23^. We did not determine RQ values directly, but took the resting RQ value of our adult male rats to be 0.9 on the basis of work by others^33,34^. Based on extensive evidence detailed by Mendoza et al.^22^, we used a RQ value of 0.9 to calculate A-a gradient throughout the blood-gas protocols on the assumption that morphine and the thiolesters do not directly affect this value, although this must be directly addressed in our protocols at some point. Here, we had both alveolar hypoventilation and ventilation-mismatch. In almost all cases, when these two phenomena occur together and are readily reversed, the cause is decreased minute ventilation leading rapidly to atelectasis.

### Antinociception protocols

#### Tail-flick latency (TFL)

The antinociceptive effects of morphine, vehicle and d-cystine diEE were assessed by tail-flick latency (TFL) test using a Tail-Flick Analgesia Meter (IITC Life Science Inc., USA) as described previously^23,35–38^. This involved minor manual restraint while positioning the tail to apply a thermal stimulus sufficient to induce a latency of tail withdrawal of about 3.0 s in all animals. Baseline TFL was tested in all rats (-20 min time-point in Fig. 7). One group of rats (79.0 ± 0.6 days of age; 338 ± 2 g body weight, n = 9 rats) received an IV injection of vehicle (saline, 100 μL/100 g body weight) and the second group (79.7 ± 0.6 days; 342 ± 2 g, n = 9 rats) received an injection of d-cystine diEE (500 μmol/kg, IV). TFL was tested in both groups 10 and 20 min later (− 10 and 0 min in Fig. 7). At 20 min post-injection (time 0), all rats received an injection of morphine (10 mg/kg, IV) and TFL tested 20, 40, 60, 90, 120, 150, 180, 210, 240, 360 and 480 min post-injection. Data are shown as actual TFL (sec) and as “maximum possible effect” (%MPE) using the formula, %MPE = [(post-injection TFL − baseline TFL)/(12 − baseline TFL)] × 100^23,35–38^.

### Antinociception assessment by Paw withdrawal Assay

#### Hot-plate latency (HPL)

The antinociceptive effects of morphine, vehicle and d-cystine diEE were assessed by hot-plate (hindpaw withdrawal) latency (HPL) test using the Hargreaves’s test^39^. In brief, paw withdrawal latency to a thermal stimulus was assessed using a radiant heat source (IITC, CA, USA) aimed at the planter surface of the left hind-paw. This method did not involve restraint while positioning the thermal stimulus sufficient enough to induce a latency of tail withdrawal of 20 s (baseline values) prior to injection of any drug (cut-off latency of 20 s was set to avoid tissue damage). Baseline HPL was tested in all rats (-20 min time-point in Fig. 7). One group of rats (80.3 ± 0.6 days of age; 340 ± 3 g body weight, n = 9 rats) received an IV injection of vehicle (saline, 100 μL/100 g body weight) and the second group (80.0 ± 0.5 days; 339 ± 3 g, n = 9 rats) received an injection of d-cystine diEE (500 μmol/kg, IV). HPL was tested in both groups 10 and 20 min later (-10 and 0 min in Fig. 7). At 20 min post-injection (time 0), all rats received an injection of morphine (10 mg/kg, IV) and HPL was tested 20, 40, 60, 90, 120, 150, 180, 210, 240, 360 and 480 min post-injection. Data are shown as actual HPL (sec) and as “maximum possible effect” (%MPE) using the formula, %MPE = [(post-injection HPL − baseline HPL)/(20 − baseline HPL)] × 100.

### Statistics

The recorded data (1 min bins) and derived parameters, Vt/Ti and Response Area (cumulative percent changes from pre-values) were taken for statistical analyses. The pre-drug 1 min bins excluded occasional marked deviations from resting due to movements or scratching by the rats. These exclusions ensured accurate determinations of baseline parameters. The data are presented as mean ± SEM. All data unless otherwise stated (see immediately below) were analyzed by one-way or two-way analysis of variance followed by Student's modified *t* test with Bonferroni corrections for multiple comparisons between means using the error mean square terms from each ANOVA^40–43^. A value of *P* < 0.05 denoted the initial level of statistical significance that was modified according to the number of comparisons between means as detailed by Wallenstein et al. (1980)^41^. The modified *t-*statistic is t = (mean group 1—mean group 2)/[s × (1/n_1_ + 1/n_2_)^1/2^] where s^2^ = the mean square within groups term from the ANOVA (the square root of this value is used in the modified t-statistic formula) and n_1_ and n_2_ are the number of rats in each group under comparison. Based on an elementary inequality called Bonferroni's inequality, a conservative critical value for the modified *t*-statistics taken from tables of *t*-distribution using a significance level of P/m, where m is the number of comparisons between groups to be performed. The degrees of freedom are those for the mean square for within group variation from the ANOVA table. In most cases, the critical Bonferroni value cannot be obtained from conventional tables of the t- distribution but may be approximated from widely available tables of the normal curve by t* = z + (z + z^3^)/4n, with n being the degrees of freedom and z being the critical normal curve value for P/m^40–43^. Wallenstein et al.^41^ first demonstrated that the Bonferroni procedure is preferable for general use since it is easiest to apply, has the widest range of applications, and gives critical values that will be lower than those of other procedures if the investigator is able to limit the number of comparisons, and that will be only slightly larger than those of other procedures if many comparisons are made. The practical application of the Bonferroni procedure first demonstrated by Wallenstein et al.^41^ has been supported and expanded upon by Ludbrook^42^ and by McHugh^43^. A value of *P* < 0.05 was taken as the initial level of statistical significance^40,41^. With respect to Supplemental Figures S4–S7, the data were analyzed by one-way ANOVA and Tukey’s least significance difference (LSD) test, with statistical differences taken as P < 0.05^40,41^.

## Results

### Ventilatory parameters

**Figure 1:**
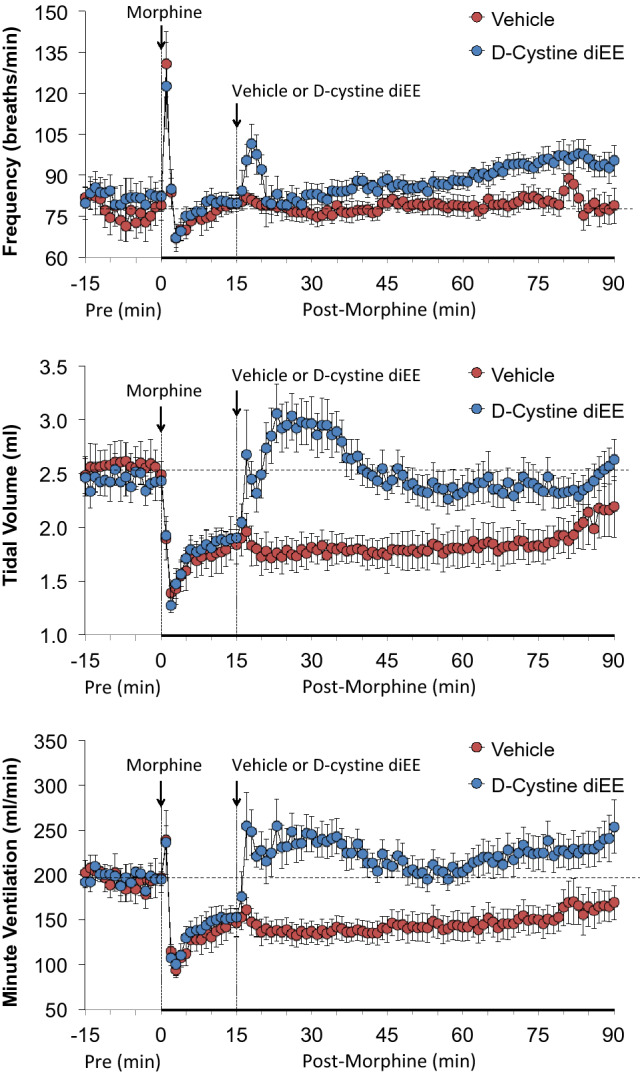
Changes in frequency of breathing (top panel), tidal volume (middle panel) and minute ventilation (bottom panel) in freely moving rats upon (a) injection of morphine (10 mg/kg, IV) and subsequent injection of vehicle (saline) or d-cystine diethyl ester (d-cystine diEE, 500 μmol/kg, IV). The data are presented as mean ± SEM. There were 9 rats in each group.

The ages and body weights of the rats and their resting ventilatory parameters prior to the commencement of the whole-body plethysmography protocols are shown in Supplemental Table S1. There were no between-group differences for any parameter (P > 0.05, for all comparisons). A summary of the maximal initial responses elicited by morphine and the total effects recorded over the 15 min prior to the injection of d-cystine diEE are summarized in Supplemental Table S2. The changes in frequency of breathing (Freq), tidal volume (TV) and minute ventilation (MV) upon injection of morphine (10 mg/kg, IV) and subsequent injection of vehicle or d-cystine diEE (500 μmol/kg, IV) are summarized in Fig. 1. The injection of morphine elicited a brief increase in Freq that was followed a relatively transient decrease that recovered before injection of vehicle of d-cystine diEE. Injection of vehicle did not elicit an immediate response in Freq, which remained at pre-injection values throughout the recording period. The injection of d-cystine diEE elicited a brief increase in Freq of about 5 min in duration that was followed by a gradual and sustained elevation in Freq. The injection of morphine elicited a prompt and sustained decrease in TV that was still pronounced at the time that vehicle of d-cystine diEE was given. Injection of vehicle did not affect TV, which gradually recovered to pre-injection levels toward the end of the recording period. As a result of the above changes in Freq and TV, it can be seen that morphine elicited a transient increase in MV that was followed by a sustained decrease and that d-cystine diEE elicited a prompt and long-lasting reversal of this effect of morphine.

**Figure 2:**
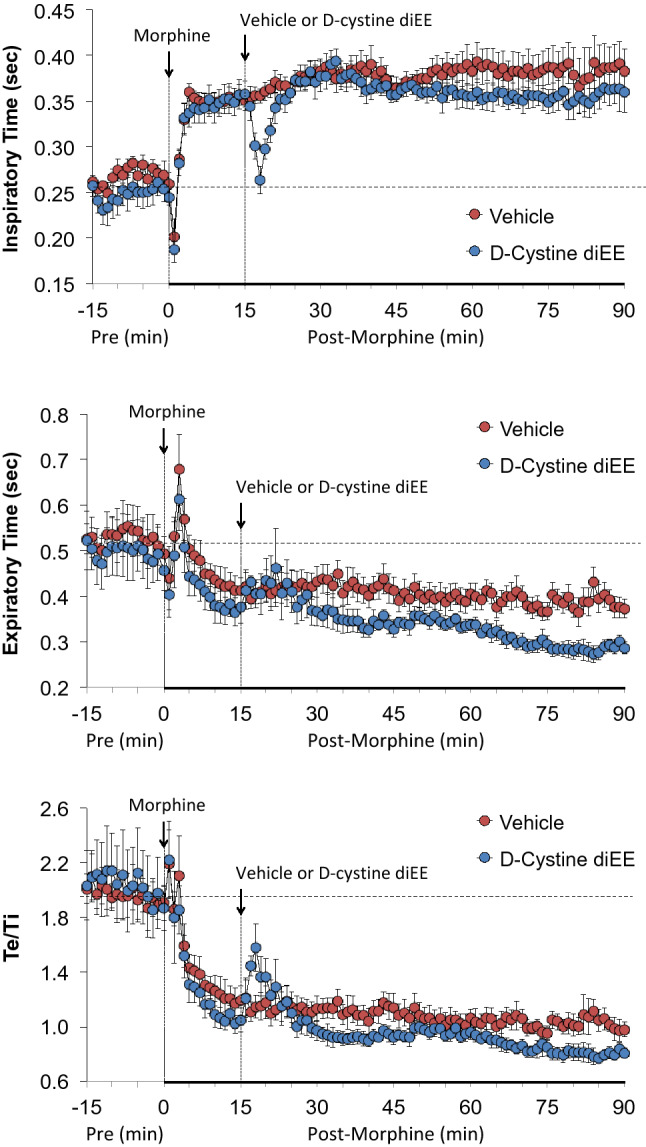
Changes in inspiratory time (top panel), expiratory time (middle panel) and expiratory time/inspiratory time (Te/Ti) (bottom panel) in freely moving rats upon (a) injection of morphine (10 mg/kg, IV) and subsequent injection of vehicle (saline) or d-cystine diethyl ester (d-cystine diEE, 500 μmol/kg, IV). The data are presented as mean ± SEM. There were 9 rats in each group.

As summarized in Fig. 2, morphine elicited a transient decrease in Ti and Te that was followed by sustained increases in Ti and decreases in Te in rats that received vehicle 15 min after injection of morphine. The injection of d-cystine diEE elicited a brief decrease in Ti without greatly affecting Te. The long-lasting increase in Ti elicited by morphine was minimally smaller in d-cystine diEE-treated rats whereas the long-lasting decrease in Te was observably greater in the presence of d-cystine diEE. The ratio of Te/Ti fell markedly after the administration of morphine in the vehicle treated rats and similarly in the d-cystine diEE-treated rats.

**Figure 3:**
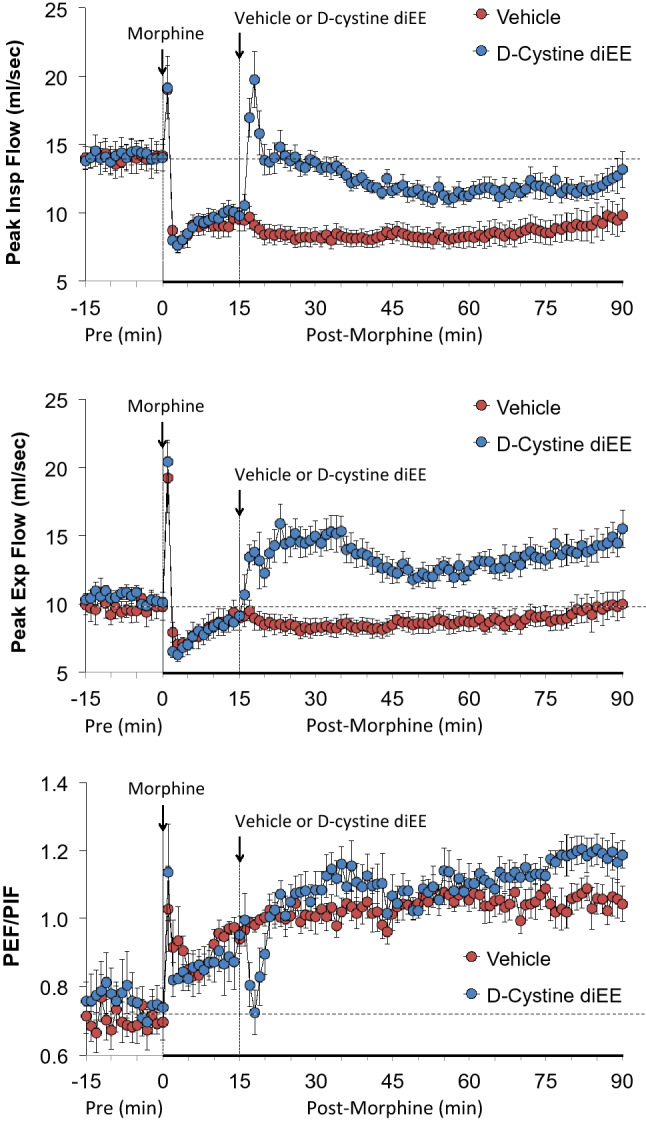
Changes in peak inspiratory flow (top panel), peak expiratory flow (middle panel) and peak expiratory flow/peak inspiratory flow (PEF/PIF) (bottom panel) in freely moving rats upon (a) injection of morphine (10 mg/kg, IV) and subsequent injection of vehicle (saline) or d-cystine diethyl ester (d-cystine diEE, 500 μmol/kg, IV). The data are presented as mean ± SEM. There were 9 rats in each group.

Figure 3 demonstrates that morphine elicited a pronounced and sustained decrease in PIF but lesser decreases in PEF in vehicle-treated rats. d-cystine diEE elicited a prompt and relatively sustained reversal of the effects of morphine on PIF and a marked increase in PEF to levels well above pre-morphine levels. Except for a decrease in PEF/PIF immediately upon injection of d-cystine diEE, the temporal changes in PEF/PIF elicited by morphine were similar in both groups.

**Figure 4:**
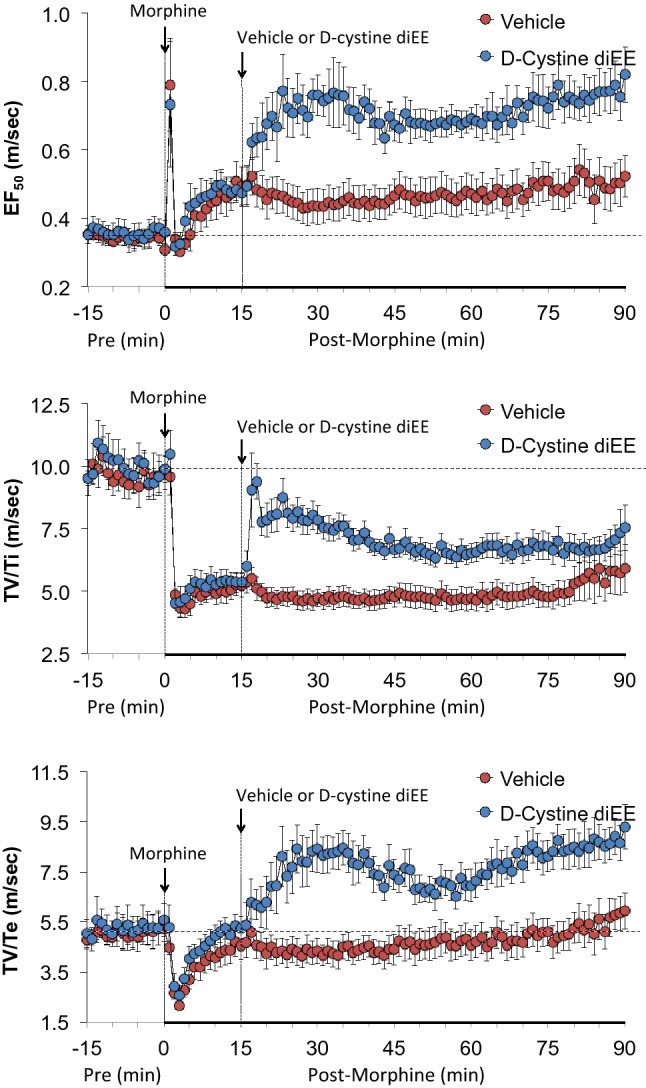
Changes in EF_50_ (top panel), inspiratory drive (TV/Ti) (middle panel) and expiratory drive (TV/Te) (bottom panel) in freely moving rats upon (a) injection of morphine (10 mg/kg, IV) and subsequent injection of vehicle (saline) or d-cystine diethyl ester (d-cystine diEE, 500 μmol/kg, IV). The data are presented as mean ± SEM. There were 9 rats in each group.

Figure 4 demonstrates that morphine elicited a sustained increase in EF_50_ in rats that received vehicle. Administration of d-cystine diEE elicited a further prompt and sustained increase in EF_50_ in morphine-treated rats. Morphine elicited a prompt and sustained decrease in inspiratory drive (TV/Ti) and relatively pronounced but shorter-lived decrease in expiratory drive (TV/Te). The injection of d-cystine diEE elicited a noticeable but partial recovery of inspiratory drive and a substantial and sustained increase in expiratory drive to well above pre-morphine levels.

The initial peak responses and the total responses elicited by vehicle or d-cystine diEE in morphine-treated rats are shown in Supplemental Figure S1. d-Cystine diEE elicited pronounced increases in Freq (along with a decrease in Ti but not Te, and an increase in Te/Ti), TV, MV, PIF and PEF (with a decrease in PE/PEF), EF_50_, and inspiratory drive (TV/Ti and expiratory drive (TV/Te). In terms of the total response, d-cystine diEE elicited a relatively minor increase in Freq and decreases in Ti and Te, but robust sustained increases in TV, MV, PIF, PEF, PEF/PIF, EF_50_ and in inspiratory drive and expiratory drives.

In contrast to d-cystine diEE, the injection of d-cystine (500 μmol/kg, IV) did not elicit immediate effects on Freq, TV or MV in morphine (10 mg/kg, IV)-treated rats although these parameters returned toward pre-morphine levels more quickly than in the vehicle-treated rats as seen in the last 15 min of the recording period (Supplemental Figure S2, Supplemental Table S3. In addition, the injection of the potent reducing agent, l-NACme (2 × 500 μmol/kg, IV), elicited only minor effects on morphine (10 mg/kg, IV)-induced changes in Freq, TV and MV (Supplemental Figure S3, Supplemental Table S3).

### Blood-gas chemistry

**Figure 5:**
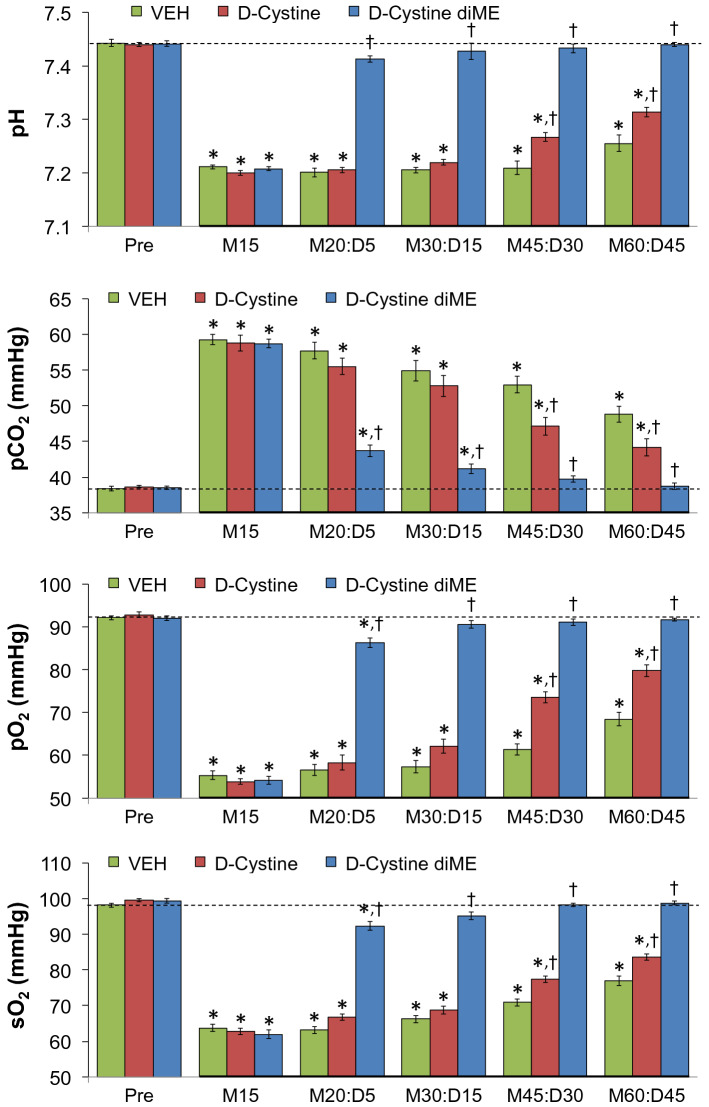
Changes in pH, pCO_2_, pO_2_ and sO_2_ elicited by injection of morphine (10 mg/kg, IV) in 3 separate groups of freely moving rats followed by injection of vehicle (VEH, saline), d-Cystine (500 μmol/kg, IV) or d-cystine dimethyl ester (d-cystine diME, 500 μmol/kg, IV). M15–M60, 15–60 min after injection of morphine. D5–D45, 5–45 min after injection of drug (vehicle, d-Cystine or d-cystine diME). The data are shown as mean ± SEM. There were 9 rats in each group. *P < 0.05, significant change from Pre-values. ^†^P < 0.05, d-cystine diEE versus vehicle.

The changes in pH, pCO_2_, pO_2_ and sO_2_ elicited by injection of morphine (10 mg/kg, IV) in 3 separate groups of freely moving rats followed by injection of vehicle (VEH, saline), d-Cystine (500 μmol/kg, IV) or d-cystine diME (500 μmol/kg, IV) are summarized in Fig. 5. The M15-M60 term on each x-axis refers to 15–60 min after injection of morphine whereas D5-D45 refers to 5–45 min after injection of drug (vehicle, d-Cystine or d-cystine diME). Morphine elicited substantial falls in pH, pO_2_ and sO_2_ accompanied by substantial increases in pCO_2_ (see time M15) Pre-values and responses to morphine were similar in the 3 groups. These values returned toward pre-injection after injection of vehicle. The values returned toward pre-injection levels faster after injection of d-cystine with these changes reaching significance at M45:D30 and M60:D45 time-points. The morphine-induced changes in ABG chemistry were reversed immediately (at M20:D5) by injection of d-cystine diME and this reversal was sustained throughout the experiment (at M60:D45). In contrast, the injection of d-cystine diME (500 μmol/kg, IV) elicited minimal immediate effects on Freq, TV and MV in morphine (10 mg/kg, IV)-treated rats.

### Alveolar-arterial gradients

**Figure 6:**
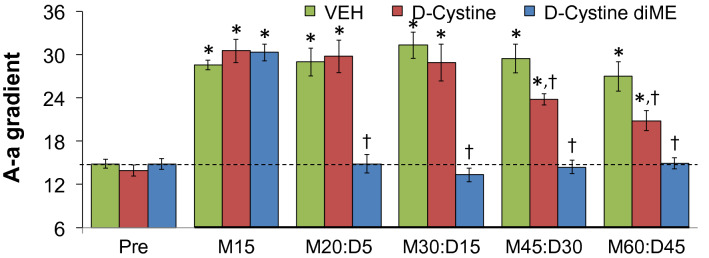
Changes in A-a gradient elicited by an injection of morphine (10 mg/kg, IV) in 3 separate groups of freely moving rats followed by injection of vehicle (VEH, saline), d-Cystine (500 μmol/kg, IV) or d-cystine dimethyl ester (d-cystine diME, 500 μmol/kg, IV). M15–M60, 15–60 min after injection of morphine. D5–D45, 5–45 min after injection of drug (vehicle, d-Cystine or d-cystine diME). The data are shown as mean ± SEM. There were 9 rats in each group. *P < 0.05, significant change from Pre-values. ^†^P < 0.05, d-cystine diEE versus vehicle.

The changes in A-a gradients in the 3 groups of freely moving rats described under *Blood-gas Chemistry* elicited by morphine (10 mg/kg, IV) and then vehicle (VEH, saline), d-Cystine (500 μmol/kg, IV) or d-cystine diME (500 μmol/kg, IV) are shown in Fig. 6. Morphine elicited substantial and equivalent increases in pCO_2_ in the 3 groups of rats (see time M15). These values did not return to pre-injection levels after injection of vehicle but returned toward pre-injection levels after injection of d-cystine, with these changes being significant at M45:D30 and M60:D45 times. Morphine-induced increases in A-a gradient were reversed immediately (at M20:D5) by d-cystine diME and this reversal was sustained throughout the experiment (at M60:D45).

### Antinociception assays

**Figure 7:**
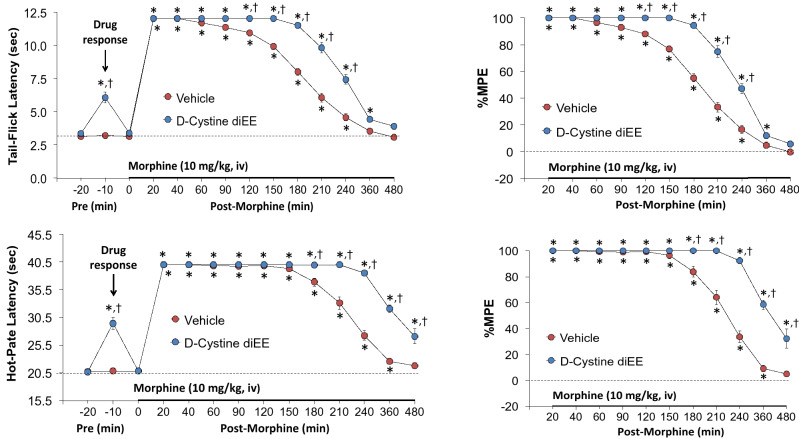
Changes in tail-flick latency (top panel) and hot-plate latency (bottom panel) elicited by injection of vehicle (VEH, saline) or d-cystine diethyl ester (d-cystine diEE, 500 μmol/kg, IV) and a subsequent injection of morphine (10 mg/kg, IV) in freely moving rats. The left-hand panels show actual data whereas the right-hand panels display the data as maximum possible effect (%MPE). The data are shown as mean ± SEM. There were 9 rats in each group. *P < 0.05, significant change from Pre. ^†^P < 0.05, d-cystine diEE versus vehicle.

The following experiments addressed the important issue as to whether the stereoisomeric configuration of cystine diME is a factor in any effects that this thiolester may have on the analgesic actions of morphine. We first tested the effects of d-cystine diME (500 μmol/kg, IV) or L-cystine diME (500 μmol/kg, IV) on analgesic status of adult male rats when given alone or when given in combination with morphine sulfate (1.0 mg/kg, IV) with testing performed between 20 to 30 min post-injection (see Supplement section S1, Effects of d-cystine diME and L-cystine diME on Antinociception Status, including Supplementary Figures S4–S7 and accompanying text and references). In brief, neither d-cystine diME nor L-cystine diME affected thermal nociception (Hargreaves Testing—heat applied to a hindpaw) or mechanical allodynia (Von Frey Testing—pressure applied to a hindpaw) when given alone and neither thiolester affected the antinociception actions of morphine. However, we wanted to further explore whether d-cystine diME would affect a higher dose of morphine and to track the changes in antinociception status over a much longer time-course. Changes in tail-flick latencies (TFL, top panel) and hot-plate latencies (HPL, bottom panel) elicited by injection of vehicle or d-cystine diEE (500 μmol/kg, IV) and subsequent injection of morphine (10 mg/kg, IV) in freely moving male rats are summarized in Fig. 7. d-cystine diEE elicited a transient increase in TFL and HPL (both effects indicative of antinociception) that resolved within 15 min (time 0). The injection morphine elicited a pronounced increase in TFL and HPL of at least 4 h in duration in vehicle-treated rats. The antinociceptive effects of morphine were enhanced in d-cystine diEE-treated rats in that the maximal possible effect (%MPE) and duration of antinociception was greater than in vehicle-treated rats from 90 min after morphine injection. The antinociceptive effects of morphine were also enhanced by d-cystine (500 μmol/kg, IV) although to a lesser degree than by d-cystine diEE (Supplemental Table S5).

### Sedation

All rats that received morphine (10 mg/kg, IV) plus vehicle remained obviously sedated (they remained on their side not moving with their eyes closed) for at approximately 60 min, after which time they gradually recovered their footing and were able to groom and move about the chamber although full mobility was not evident for at least 2 h. Sedation in the rats that received morphine plus d-cystine diEE or d-cystine diME was indistinguishable from the rats the received morphine plus vehicle. The durations of the sedative and analgesic actions of morphine far exceeded the ventilatory depression elicited by the opioid (see Figs. 1, 2, 3, 4, 5, 6, 7).

## Discussion

The present study demonstrates that the systemic injection of d-cystine diEE or d-cystine diME elicit an immediate and sustained reversal of the negative effects of a 10 mg/kg dose of morphine on ventilatory parameters, gas exchange in the lungs (elevated A-a gradient) and ABG chemistry in unanesthetized adult male Sprague–Dawley rats without (apparently) affecting the sedative effects of morphine and while augmenting the antinociceptive effects of the opioid. The major findings with respect to potential clinical impact are clearly that d-cystine diEE or d-cystine diME reversed the negative effects of morphine on MV and ABG chemistry, effects that would be the major contributors to restoration of ventilatory performance. The ability of d-cystine diEE or d-cystine diME to reverse the other effects of morphine such as depression of PIF, while important must be considered secondary to the effects on MV and especially the TV component. Taken together, it would appear unlikely that d-cystine diEE or d-cystine diME directly modulate the pharmacological actions of morphine by competitive or non-competitive blockade of opioid receptors since all of the above effects of morphine are antagonized by opioid receptor antagonists such as naloxone and naltrexone^1–5^. The site(s) of action and molecular mechanisms by which d-cystine diEE or d-cystine diME exert their robust effects on ventilatory parameters, A-a gradient and ABG chemistry in morphine-treated rats while augmenting the antinociceptive effects of the opioid, remain to be determined. Evidence that morphine blocks the entry of l-cysteine into neurons via inhibition of EAA3^6,7^ raises the possibility that either (a) a decrease in intracellular levels of l-cysteine and resulting enhancement of the oxidative (less reductive) status of the cell^6,7^ and/or (b) loss of participation of l-cysteine in a myriad of intracellular metabolic pathways including the generation of the gaseous neurotransmitter hydrogen sulfide^44–46^, plays a role in the deleterious actions of morphine while conversely promoting the antinociceptive and sedative actions of the opioid.

The potent actions of d-cystine diEE, d-cystine diME and l-cysteine ethyl ester^22^ on the negative effects of morphine on ventilation and gas-exchange support these concepts whereas the ability of the thiolesters to augment the antinociceptive actions of morphine does not. Transport of cystine esters into the cell would not itself correct for the loss of sulfhydryl equivalents since cystine is already in the more oxidized disulfide state, and d-cystine or D-cysteine would not participate in most of the metabolic pathways of l-cysteine, but uptake of d-cystine esters could potentially drive up levels of intracellular l-cysteine. However, our finding that the highly cell-permeable thiolester reducing agent, N-acetyl-l-cysteine methyl ester^21^, had minimal effects on the ventilatory depressant effects of morphine suggests that d-cystine diEE and d-cystine diME do not act simply by increasing reducing equivalents in cells. Potential mechanisms of action of d-cystine diEE and d-cystine diME may involve (a) interference with opioid receptor-linked β-arrestin cell signaling, which would spare the Gprotein-mediated antinociceptive actions of morphine^47,48^, and/or conversion of these thiolesters to bioactive S-nitrosothiols (i.e., S-nitroso- d-cystine diEE, S-nitroso- d-cystine diME) that may act as intracellular nitrosating agents similar to S-nitroso-l-cysteine ethyl ester (28, 29)^49,50^. S-nitrosothiols in the brainstem, peripheral structures and red blood cells play important roles in ventilatory control processes^51–55^. For example, microinjection of S-nitrosothiols into the nucleus tractus solitarius elicit robust increases in MV^54^ as do systemic delivery of S-nitrosothiols to the carotid bodies^55^. Our evidence that S-nitrosothiols such as S-nitroso-l-cysteine exert their ventilatory effects via direct modulation of voltage-gated K^+^-channels may represent a molecular target for d-cystine diEE and d-cystine diME and their S-nitrosothiol forms, which may target the intracellular domains of these channels.

As we reported previously^24–27^, the 10 mg/kg dose of morphine elicited only a transient decrease in Freq. This apparent lack of sustained effects on Freq is misleading in the sense that morphine elicited a profound and sustained increase in Ti and a sustained decrease in Te (present study)^24–27^. Despite evidence that the depressant effects of morphine on Freq involve suppression of carotid body chemoreceptor reflexes^27^, we reported that the ventilatory depressant effects of morphine (10 mg/kg, IV) in freely moving rats were exacerbated in rats with bilateral carotid sinus nerve transection^27^, suggesting that morphine does not directly affect or potentially promotes carotid body chemoreflexes in these unanesthetized rats. d-cystine diEE had minor effects on the actions of morphine on the above parameters (i.e., Freq rose to higher levels than in vehicle-treated rats, whereas Ti did not rise as much and Te deceased to a greater extent). It would seem that the carotid body may not be a major site of direct action considering the minimal effects of the thiolester on Freq.

The first novel set of findings in the present study was that d-cystine diEE elicited an immediate and sustained reversal of the negative effects of morphine on TV (and therefore MV), PIF, PEF, and inspiratory and expiratory drives, while promoting the enhancing the effects of morphine on EF_50_. In contrast, the injection of the parent thiol, d-cystine, did not elicit immediate responses in morphine-treated rats, although Freq, TV and MV (and other variables, data not shown) returned to pre-morphine levels somewhat more rapidly than in vehicle-injected rats. The second novel set of findings was that d-cystine diME elicited an immediate and sustained reversal of the negative effects of morphine on ABG chemistry whereas d-cystine produced a gradual recovery that was greater than in vehicle-injected rats. This is related to the third novel finding that d-cystine diME elicited a prompt and sustained reversal of the negative effects of morphine on gas-exchange within the lungs (as defined by reversal of the morphine-induced increase in A-a gradient) whereas again, d-cystine promoted the recovery of the effects of morphine from about 30 min after the injection of the thiolester (45 min post-morphine). Taken together, the ability of d-cystine diEE/diME to reverse the above negative effects of morphine is due to a unique profile of activity that also includes potentiation of the antinociceptive actions of the opioid. With respect to antinociception, the ability of systemically injected d-cystine diEE to elicit a transient antinociception (as detected by both TF and HP assays) is consistent to a degree with evidence that direct injection of d-cystine into the hindpaw of rats elicited profound blockade of thermal nociception^56^. d-cystine may exert its effects on nociceptive processing via redox modulation (closure) of ion-channels such as T-type voltage-gated Ca^2+^ channels^57^. Moreover, Lee et al.^58^ demonstrated that extracellular application of S-nitrosothiols such as S-nitrosoglutathione rapidly reduced T-type Ca^2+^ current amplitudes in sensory cell bodies within dorsal root ganglia. Whether any of these actions participate in d-cystine diEE-induced promotion of the antinociceptive effects of morphine remains to be determined. Obviously numerous processes such as alterations in opioid receptor status (e.g., desensitization, phosphorylation, internalization) and promotion of the intracellular cascades by which opioids induce antinociception may be involved^59,60^.

The gradual appearance of effects of d-cystine on morphine-induced changes in ventilatory parameters, ABG chemistry, A-gradient and TFL raises the possibility that d-cystine diEE and d-cystine diME exert their effects via rapid introduction of d-cystine into cells as opposed to gradual entry of d-cystine through uptake systems. The uptake of L-cystine into cells is mediated by the cystine-glutamate antiporter system x^c-^ and the Na^+^-independent high-affinity cystine transporter, b0, + AT^61–63^. There is evidence that cystine-glutamate antiporter system x^c-^ does not transport d-cystine^62^ and to our knowledge it is not known whether d-cystine is transported by b0, + AT. As such, the mechanisms (e.g., facilitated entry via transporters, conversion to other compounds which gain cell entry or act on membrane proteins), by which d-cystine exerts its latent effects remain unknown, but are worthy of examination.

## Conclusion

We present evidence here that d-cystine diEE, d-cystine diME and to a lesser degree, d-cystine itself, represent a novel class of compounds that have an important therapeutic profile that may be of value in the clinic to treat opioid-induced respiratory depression without compromising antinociception. Taken together, it is evident that d-cystine diEE and d-cystine diME are able to impair some of the actions of morphine (i.e., OIRD) but not others (i.e., antinociception, sedation). It would therefore appear that the thiolesters do not directly interfere with opioid receptors but that the delivery of thiolesters to neurons participating in OIRD may differentially affect the signaling processes (e.g., Gprotein- and β-arrestin-dependent)^47,48^ that mediate the effects of morphine on breathing. The positive results of d-cystine diEE against morphine in the ventilation (plethysmography) studies coupled to the positive effects of d-cystine diME against morphine in the ABG chemistry and A-a gradient studies would suggest that while the presence of an ester linkage is vital to cell penetration, whether this linkage is an ethyl ester or methyl ester may not be a key determinant of bioactivity against morphine. In addition, the lack of effect of the powerful reducing agent l-NACme^21^ on morphine-induced respiratory depression would tentatively argue that the effects of d-cystine diEE and d-cystine diME are not simply due to the breakdown of the disulfide esters into the monothiol (reduced) forms, which then exert their intracellular actions as reducing agents. We have established methods to determine the pharmacokinetic profile of d-cystine diME in dog plasma^64^ and developed an ultra-sensitive method to detect trace concentrations of S-nitrosothiols by means of a capacitive sensor^65^. It will be of considerable interest to see whether the pharmacokinetics of d-cystine diEE and d-cystine diME are altered meaningfully by opioids and whether thiolesters generate and/or are converted to S-nitrosothiols.

The issue as to whether the ability of thiolesters to alter redox status of neurons/cells involved in overcoming OIRD certainly needs to be addressed in future studies. It has been demonstrated that oral ingestion of the antioxidant, N-acetyl-cysteine, can improve the hypoxic ventilatory response in humans^66^, and that a combination of the antioxidants, ascorbic acid and α-tocopherol, reverse the decrease in the hypoxic ventilatory response induced by acetazolamide^67^ or the sub-anesthetic administration of isoflurane^68^ in healthy humans. Our future studies will explore the ability of the above mentioned ascorbic acid and α-tocopherol combination in particular to reverse OIRD in rats since the lack of effect of l-NACme in our study would suggest that N-acetyl-cysteine may not be effective at least when given as an acute injection.

## Supplementary Information

Supplementary Information
