# Supplementary material for: d-Cystine di(m)ethyl ester reverses the deleterious effects of morphine on ventilation and arterial blood gas chemistry while promoting antinociception
Source: Sci Rep. 2021 May 11;11:10038. doi: 10.1038/s41598-021-89455-2 (PMC8113454; doi:10.1038/s41598-021-89455-2)
Supplement: Supplementary file 1 — Supplementary Information [file 41598_2021_89455_MOESM1_ESM.docx]

**Supplemental**

**D-Cystine di(m)ethyl ester reverses the deleterious effects of morphine on ventilation and arterial blood gas chemistry while promoting antinociception**

Benjamin Gaston^1^, Santhosh M. Baby^9^, Walter J. May^2^, Alex P. Young^2^, Alan Grossfield^3^,

James N. Bates^4^, James M. Seckler^5^, Christopher G. Wilson^6^ & Stephen J. Lewis^7,8,^*

*^1^Herman B Wells Center for Pediatric Research, Indiana University School of Medicine,*

*Indianapolis, IN 46202, USA.*

*^2^Pediatric Respiratory Medicine, University of Virginia School of Medicine,*

*Charlottesville, VA 22908, USA.*

*^3^Department of Biochemistry and Biophysics, University of Rochester Medical Center,*

*Rochester, NY 14642, USA.*

*^4^Department of Anesthesia, University of Iowa Hospitals and Clinics, Iowa City, IA 52242, USA. ^5^Department of Biomedical Engineering, Case Western Reserve University*

*Cleveland, OH 44106, USA.*

*^6^Basic Sciences, Division of Physiology, School of Medicine, Loma Linda University,*

*Loma Linda, CA 92350, USA.*

*^7^Department of Pharmacology, Case Western Reserve University, Cleveland, OH 44106, USA.*

*^8^Division of Pulmonology, Allergy and Immunology, Departments of Pediatrics, School of Medicine, Case Western Reserve University, 10900 Euclid Avenue, Cleveland, OH 44106-4984, USA.*

*^9^Present address: Translational Sciences Treatment Discovery, Galvani Bioelectronics, Inc., 1250 S Collegeville Rd., Collegeville, PA 1r9426, USA*

***Corresponding Author**

Stephen J. Lewis, PhD. Department of Pediatrics, Division of Pulmonology, Allergy and Immunology, School of Medicine, Case Western Reserve University, 10900 Euclid Avenue, Cleveland, OH 44106-4984. Phone: 216-368-3482. Email: sjl78@case.edu

**Conflict of interest statement**

The authors have declared that no conflict of interest exists

**Supplemental Table 1.** Baseline (pre) values in the groups of rats that would receive vehicle of D-cystine diEE.

| **Parameter** |  | **Vehicle** |  | **D-Cystine diEE** |
| --- | --- | --- | --- | --- |
| Age, days |  | 78.3 ± 0.4 |  | 78.0 ± 0.6 |
| Body weights, gram |  | 338 ± 2 |  | 337 ± 2 |
| Frequency, breaths/min |  | 79.5 ± 4.5 |  | 83.7 ± 5.4 |
| Tidal Volume (TV), ml |  | 2.53 ± 0.17 |  | 2.44 ± 0.13 |
| Minute Ventilation, ml/min |  | 198 ± 14 |  | 200 ± 12 |
| Inspiratory Time (Ti), sec |  | 0.261 ± 0.012 |  | 0.240 ± 0.010 |
| Expiratory Time (Te), sec |  | 0.503 ± 0.032 |  | 0.476 ± 0.051 |
| Inspiratory Time/Expiratory Time |  | 1.93 ± 0.12 |  | 2.03 ± 0.25 |
| Peak Inspiratory Flow, ml/sec |  | 14.1 ± 0.9 |  | 14.2 ± 0.8 |
| Peak Expiratory Flow, ml/sec |  | 10.1 ± 0.7 |  | 10.5 ± 0.3 |
| Peak Expiratory Flow/Peak Inspiratory Flow |  | 0.73 ± 0.03 |  | 0.77 ± 0.06 |
| EF_50_, ml/sec |  | 0.34 ± 0.02 |  | 0.37 ± 0.02 |
| Inspiratory Drive (TV/Ti), ml/sec |  | 9.7 ± .7 |  | 10.3 ± 0.3 |
| Inspiratory Drive (TV/Te), ml/sec |  | 5.2 ± 0.4 |  | 5.5 ± 0.5 |

D-Cystine diEE, D-cystine diethyl ester. The data are presented as mean ± SEM. There were 9 rats in each group. There were no between group differences for any parameter (P > 0.05, for all comparisons.

**Supplemental Table 2. Morphine-induced ventilatory responses in rats that subsequently received vehicle or D-Cystine diEE**

|  |  | **Peak Responses (%change)** | | |  | **Total Response (% change)** | | |
| --- | --- | --- | --- | --- | --- | --- | --- | --- |
| **Parameters** |  | **Vehicle** |  | **D-Cystine diEE** |  | **Vehicle** |  | **D-Cystine diEE** |
| Frequency, breaths/min |  | -21.5 ± 3.4* |  | -20.0 ± 4.5* |  | +0.3 ± 4.3 |  | -1.9 ± 6.6 |
| Tidal Volume (TV), ml |  | -42.9 ± 8.7* |  | -35.8 ± 6.9* |  | -29.3 ± 7.6* |  | -26.3 ± 7.4* |
| Minute Ventilation, ml/min |  | -51.2 ± 6.3* |  | -48.5 ± 6.0* |  | -28.6 ± 7.2* |  | -28.4 ± 6.4* |
| Inspiratory Time (Ti), sec |  | +37.9 ± 3.0* |  | +41.3 ± 7.1* |  | +38.6 ± 2.9* |  | +40.2 ± 6.3* |
| Expiratory Time (Te), sec |  | -29.3 ± 17.1 |  | -32.4 ± 17.6 |  | -4.7 ± 6.0 |  | -2.0 ± 6.1 |
| Inspiratory Time/Expiratory Time |  | -38.0 ± 4.2* |  | -41.7 ± 7.8* |  | -24.1 ± 4.7* |  | -27.1 ± 6.2* |
| Peak Inspiratory Flow, ml/sec |  | -45.6 ± 7.0* |  | -43.2 ± 2.7* |  | -29.5 ± 8.1* |  | -29.7 ± 2.7* |
| Peak Expiratory Flow, ml/sec |  | -38.1 ± 8.5* |  | -34.8 ± 6.7* |  | -17.4 ± 6.1* |  | -16.2 ± 5.7* |
| Peak Expiratory Flow/Peak Inspiratory Flow |  | +30.2 ± 3.4* |  | +26.7 ± 5.4* |  | +27.6 ± 7.0* |  | +21.5 ± 6.7* |
| EF_50_, ml/sec |  | +133 ± 42* |  | +122 ± 27* |  | +30.5 ± 14.7* |  | +33.4 ± 15.0* |
| Inspiratory Drive (TV/Ti), ml/sec |  | -52.7 ± 5.4* |  | -53.7 ± 6.0* |  | -44.3 ± 7.1* |  | -44.5 ± 5.5* |
| Expiratory Drive (TV/Te), ml/sec |  | -51.5 ± 10.1* |  | -46.2 ± 7.8* |  | -22.3 ± 13.6 |  | -14.6 ± 10.2 |

D-Cystine diEE, D-cystine diethyl ester (500 μmol/kg, IV). The data are presented as mean ± SEM. There were 9 rats in each group. There were no between group differences for any parameter (P > 0.05, for all comparisons. *P < 0.05, D-Cystine diEE versus vehicle.

**Supplemental Figure 1**

**Peak response elicited by vehicle or D-Cystine diEE**

**Total responses elicited by vehicle or D-Cystine diEE**

**Supplemental Figure 1. Top panel:** Peak percent changes in ventilatory parameters elicited by injection of vehicle or D-cystine diethyl ester (D-cystine diEE, 500 μmol/kg, IV) in separate groups of morphine (10 mg/kg, IV)-treated rats. Bottom panel: Total percent changes in ventilatory parameters elicited by injection of vehicle or D-cystine diethyl ester (D-cystine diEE, 500 μmol/kg, IV) in separate groups of morphine (10 mg/kg, IV)-treated rats. The data are shown as mean ± SEM. There were 9 rats in each group. *P < 0.05, significant change from Pre-values. ^†^P < 0.05, D-cystine diEE *versus* vehicle.

**Supplemental Figure 2**

**Supplemental Figure 2.** Changes in frequency of breathing (top panel), tidal volume (middle panel) and minute ventilation (bottom panel) in freely moving rats upon (a) injection of morphine (10 mg/kg, IV) and subsequent injection of vehicle (saline) or D-cystine (500 μmol/kg, IV). The data are presented as mean ± SEM. There were 9 rats in each group.

**Supplemental Table 3.** Morphine-induced responses in rats that subsequently received vehicle or D-Cystine

|  |  |  |  | **Treatment Groups** | | |
| --- | --- | --- | --- | --- | --- | --- |
| **Parameters** |  | **Stage of Experiment** |  | **Vehicle** |  | **D-Cystine** |
| **Number** |  | Morning of experiment |  | 9 |  | 9 |
| **Age, days** |  | Morning of experiment |  | 79.7 ± 0.4 |  | 80.0 ± 0.5 |
| **Body weights, g** |  | Morning of experiment |  | 340 ± 2 |  | 341 ± 3 |
| **Frequency** |  | Pre values |  | 82 ± 5 |  | 81 ± 6 |
|  |  | Morphine - peak (+) response (%) |  | +57 ± 8* |  | +62 ± 7* |
|  |  | Morphine peak (-) response (%) |  | -21 ± 3* |  | -23 ± 4* |
|  |  | Morphine - first 15 min response (%) |  | -3.8 ± 2.6 |  | -4.7 ± 3.1 |
|  |  | Drug maximum, %change |  | -2.4 ± 1.3 |  | -1.2 ± 2.1 |
|  |  | Drug - entire 75 min, %change |  | +1.7 ± 0.9 |  | +4.5 ± 3.3 |
|  |  | Drug – last 15 min, %change |  | +2.0 ± 0.9* |  | +14.3 ± 2.8*^,†^ |
| **Tidal Volume** |  | Pre values |  | 2.59 ± 0.15 |  | 2.52 ± 0.14 |
|  |  | Morphine - peak (+) response (%) |  | -32 ± 5* |  | -30 ± 4* |
|  |  | Morphine peak (-) response (%) |  | -49 ± 5* |  | -51 ± 6* |
|  |  | Morphine – first 15 min response (%) |  | -37 ± 6* |  | -35 ± 5* |
|  |  | Drug maximum, %change |  | +1.8 ± 0.9 |  | +3.4 ± 2.2 |
|  |  | Drug - entire 75 min, %change |  | -30 ± 5* |  | -22 ± 4* |
|  |  | Drug – last 15 min, %change |  | -24 ± 4* |  | -12 ± 2*^,†^ |
| **Minute Ventilation** |  | Pre values |  | 214 ± 15 |  | 207 ± 14 |
|  |  | Morphine - peak (+) response (%) |  | +9 ± 3* |  | +14 ± 3* |
|  |  | Morphine peak (-) response (%) |  | -60 ± 9* |  | -61 ± 8* |
|  |  | Morphine – first 15 min response (%) |  | -39 ± 4* |  | -38 ± 5* |
|  |  | Drug maximum, %change |  | -0.7 ± 1.3 |  | -4.4 ± 2.5 |
|  |  | Drug - entire 75 min, %change |  | -29 ± 4* |  | -19 ± 5* |
|  |  | Drug – last 15 min, %change |  | -23 ± 4* |  | -2 ± 4*^,†^ |

Drug refers to an injection of vehicle or D-cystine (500 μmol/kg, IV). The data are presented as mean ± SEM. There were 9 rats in each group. There were no between group differences for any Pre-value (P > 0.05, for all comparisons. *P < 0.05, significant response. ^†^P < 0.05, value in the D-cystine group *versus* value in the vehicle group.

**Supplemental Figure 3**

**Supplemental Figure 3.** Changes in frequency of breathing (top panel), tidal volume (middle panel) and minute ventilation (bottom panel) in freely moving rats upon (a) injection of morphine (10 mg/kg, IV) and two subsequent injections of vehicle (saline) or N-acetyl-L-cysteine ethyl ester (L-NACme; 500 μmol/kg, IV). The data are shown as mean ± SEM. There were 9 rats in each group.

**Supplemental Table 4.** Ventilatory responses elicited by morphine and subsequent injections of vehicle or N-acety**l**-L-cysteine dimethyl ester (L-NACme)

|  |  |  |  | **Treatment Groups** | | |
| --- | --- | --- | --- | --- | --- | --- |
| **Parameters** |  | **Stage of Experiment** |  | **Vehicle** |  | **L-NACme** |
| **Number** |  | Morning of experiment |  | 9 |  | 9 |
| **Age, days** |  | Morning of experiment |  | 80.0 ± 0.5 |  | 79.7 ± 0.4 |
| **Body weights, g** |  | Morning of experiment |  | 337 ± 3 |  | 336 ± 2 |
| **Frequency** |  | Pre values |  | 99 ± 3 |  | 98 ± 4 |
|  |  | Morphine - peak (+) response (%) |  | +47 ± 6* |  | +42 ± 6* |
|  |  | Morphine peak (-) response (%) |  | -24 ± 3* |  | -22 ± 4* |
|  |  | Morphine - first 15 min response (%) |  | -4.0 ± 2.1 |  | -4.9 ± 2.8 |
|  |  | Drug - entire 75 min, %change |  | +18 ± 4* |  | +11 ± 2* |
| **Tidal Volume** |  | Pre values |  | 2.62 ± 0.13 |  | 2.61 ± 0.16 |
|  |  | Morphine - peak (+) response (%) |  | -19 ± 3* |  | -16 ± 6* |
|  |  | Morphine peak (-) response (%) |  | -38 ± 5* |  | -40 ± 6* |
|  |  | Morphine - first 15 min response (%) |  | -33 ± 4* |  | -34 ± 3* |
|  |  | Drug - entire 75 min, %change |  | -23 ± 4* |  | -22 ± 3* |
| **Minute Ventilation** |  | Pre values |  | 256 ± 6 |  | 259 ± 17 |
|  |  | Morphine - peak (+) response (%) |  | +19 ± 5* |  | +20 ± 6* |
|  |  | Morphine peak (-) response (%) |  | -53 ± 5* |  | -54 ± 4* |
|  |  | Morphine – first 15 min response (%) |  | -36 ± 3* |  | -35 ± 3* |
|  |  | Drug - entire 75 min, %change |  | -15 ± 2* |  | -19 ± 4* |

Drug refers to two injections of vehicle or N-acetyl-L-cysteine ethyl ester (L-NACme; 500 μmol/kg, IV). The data are presented as mean ± SEM. There were 9 rats in each group. There were no between group differences for any Pre-value (P > 0.05, for all comparisons. *P < 0.05, significant response. ^†^P < 0.05, value in the D-cystine group *versus* value in the vehicle group.

**Supplemental Table 5.** Tail-Flick latency values elicited by morphine and subsequent injections of vehicle or D-Cystine.

|  |  | **Tail-Flick latency (sec)** | |
| --- | --- | --- | --- |
| **Phase** | **Time (min)** | **Vehicle** | **D-Cystine** |
| Pre | -20 | 2.8 ± 0.3 | 2.9 ± 0.3 |
| 10 min-post drug | -10 | 2.9 ± 0.3 | 3.3 ± 0.4 |
| 20 min post-drug | 0 | 2.9 ± 0.2 | 3.2 ± 0.4 |
| post-morphine - 30 min | 30 | 12 ± 0.0* | 12 ± 0.0 |
| post-morphine - 60 min | 60 | 12 ± 0.0* | 12 ± 0.0 |
| post-morphine - 90 min | 90 | 11.7 ± 0.2* | 12 ± 0.0 |
| post-morphine – 120 min | 120 | 11.1 ± 0.2* | 12 ± 0.0*^,†^ |
| post-morphine – 180 min | 180 | 8.3 ± 0.3* | 10.7 ± 0.3*^,†^ |
| post-morphine – 210 min | 210 | 5.6 ± 0.3* | 7.4 ± 0.3*^,†^ |
| post-morphine – 240 min | 240 | 4.3 ± 0.3* | 5.0 ± 0.3* |
| post-morphine - 360 min | 360 | 3.3 ± 0.3 | 3.5 ± 0.3 |

The dose of D-cystine was 500 μmol/kg, IV. The data are presented as mean ± SEM. There were 9 rats in each group. There were no between group differences for any Pre-value and neither vehicle or D-cystine elicited immediate effects as measured 10 and 20 min post-injection (P > 0.05, for all comparisons. *P < 0.05, significant difference from Pre-values. ^†^P < 0.05, D-cystine *versus* vehicle.

**Effects of D-cystine diME and L-cystine diME on antinociception status**

**Antinociception Protocols**

We used 28 rats (male, retired breeders, Harlan Laboratories, 420-480 gram body weights) with jugular catheters implanted for intravenous injections. All rats were habituated to the testing chambers (> 1h) prior to days when the drugs were tested. Each rat served as its own control (saline injection, 500 µL) and was then tested with D-cystine diME dihydrochloride (500 µmol/kg, IV), L-cystine diME dihydrochloride (500 µmol/kg, IV) or morphine sulfate (1 mg/kg, IV) alone and in combination with D-cystine diME or L-cystine diME (doses as above). For trials with morphine + D-cystine diME or morphine + L-cystine diME, we injected either morphine or the thiolesters first, waited 15 min, then injected the other compound to test for serial dependency. The antinociception testing was done 20 to 30 min after the last injection was given. All rats that received both drugs were tested on different days for the second set of injections (e.g. if a rat had received D-cystine diME first on day one, in the next trial we injected morphine first). All compounds were injected in 500 µL boli. After injections, all rats were allowed to rest in the testing chamber for approximately 20 min before beginning each trial. Data were analyzed by one-way ANOVA and Tukey’s least significance difference (LSD) test, with statistical differences taken as P < 0.05.

**Thermal Nociception (Hargreaves Testing)**

Thermal nociception was assessed using Hargreaves withdrawal test to thermal noxious stimuli (reference S1) The Hargreaves hindpaw withdrawal test is similar in principle and purpose to the tail-flick withdrawal test but has the important advantage of targeting regions of high sensory receptor density on the rat’s paws (here we used the plantar surface of the hindpaws) and shows greater reproducibility and lower variability than the tail-flick withdrawal test (S2). Briefly, the rats were placed in a Plexiglas enclosure that rests on an elevated glass platform (Plantar Test, UGO BASILE, Biological Research Apparatus, Comerio, Italy). After allowing the rats to acclimate to the chamber, a movable, focused infrared emitter was placed under the rat’s hindpaw. Left and right hindpaws were alternately tested for each successive trial; no less than 1 min passed before the opposite hindlimb was tested and at least 5 min passed before tests were performed on the same hindlimb in a given animal to minimize the effects of sensory sensitization/habituation.

A photocell automatically turned the emitter off when the animal moved its paw and the latency time for the animal to withdraw its paw is recorded. Strength of stimulation is adjusted to produce hindpaw baseline latencies of 12 to 15 sec (approximately 50-60° C). A safety cutoff of 20 sec was used to prevent prolonged exposure to the noxious heat and minimize sensitization or habituation. Five different trials are performed per paw. Because testing season, climate (humidity and temperature), time of day, the cage density, animal weight, gender, locomotor behavior, the number of instrument operators, and order of testing have a significant impact on the results of pain studies in rodents (S3-S6), we controlled these variables by performing all experiments in the Behavioral Testing Core facility at Loma Linda University which is in a light-controlled, temperature and humidity regulated room within the animal care facility.

The repetitive nature of the Hargreave’s test makes it quite susceptible to plastic changes, including learning and habituation (S7), which could introduce unwanted confounding effects and affect outcomes of sensory results (S8-S10). To facilitate data interpretation, the thermal latencies can be represented as percent change from the saline/control animals as baseline and normalized. Here, we report the raw latencies (Hargreave’s test) and forces (von Frey test) because variability in the data was low and showed high consistency between trials for each animal over time.

**Mechanical Allodynia (Von Frey Testing)**

To further assess sensory function, we also measured the withdrawal threshold of the forepaws and hindpaws in response to a mechanical stimulus using an electronic von Frey aesthesiometer (model 2391C; IITC Life Science, Woodland Hills, CA). Each rat was placed in a Plexiglas chamber positioned on an elevated metallic grid/screen, which provided access to the plantar surface of the hind paw. rats are allowed to acclimate for 30 min before testing. To perform the test, a rigid blunt tip attached to the meter and then applied to the plantar surface from underneath the grid. Withdrawal threshold is the average force (g) required for paw removal in five trials separated by a 1 min interval. Antinociception testing was done 20 to 30 min after the last injection was given.

**Results**

**L-cystine diME**

**Hargreaves Hindpaw Latency Test – see Supplemental Figure 4.** We tested 8 rats for saline and L-cystine diME, and 6 rats each for morphine + L-cystine diME and L-cystine diME + morphine. The latencies (mean ± SEM) in seconds for each treatment group are: Saline = 13.4 ± 0.7; L-cystine diME = 12.0 ± 0.5; morphine = 19.5 ± 0.4; morphine + L-cystine diME = 17.2 ± 0.9; L-cystine diME plus morphine = 18.5 ± 1.1. Morphine elicited a significant increase in withdrawal latency. The increases in latency elicited by morphine + L-cystine diME, and L-cystine diME + morphine were similar to those elicited by morphine alone (P > 0.05, for both comparisons).

**Supplemental Figure 4.** Paw-withdrawal latencies (Hargreaves test) in rats pretreated with saline (n = 8 rats), L-cystine diME (500 µmol/kg, IV; n = 8 rats), morphine sulfate (1 mg/kg, IV, n = 8 rats) and a combination of morphine plus L-cystine diME (n = 6 rats) or L-cystine diME plus morphine (n = 6 rats). The data are presented as mean ± SEM. *P < 0.05, morphine alone *versus* saline; morphine plus L-cystine diME and L-cystine diME plus morphine *versus* L-cystine diME alone.

**Von Frey Test – see Supplemental Figure 5.** Using the same test groups above, we found that the forces (g) needed to initiate hindpaw withdrawal in each group was (mean ± SEM): saline = 47.4 ± 3.3; L-Cystine diME = 51.0 ± 7.6; morphine = 88.3 ± 7.0; morphine + L-Cystine diME = 86.9 ± 5.2; L-Cystine diME + morphine = 87.3 ± 4.2. Morphine elicited a significant increase in withdrawal latency. The increases in latency elicited by morphine plus L-cystine diME, and L-cystine diME plus morphine were similar to those elicited by morphine alone (P > 0.05, for both comparisons).

**Supplemental Figure 5.** Paw-withdrawal latencies (Von Frey test) in rats pretreated with saline (n = 8 rats), L-cystine diME (500 µmol/kg, IV; n = 8 rats), morphine (1 mg/kg, IV, n = 8 rats) and a combination of morphine + L-cystine diME (n = 6 rats) or L-cystine diME + morphine (n = 6 rats). Data are presented as mean ± SEM. *P < 0.05, morphine alone *versus* saline; morphine + L-cystine diME or L-cystine diME + morphine *versus* L-cystine diME alone.

**D-cystine diME**

**Hargreaves Hindpaw Latency Test – see Supplemental Figure 6.** We tested 3 rats for D-Cystine diME, 3 rats each for morphine alone and morphine + D-Cystine diME. The latencies (mean ± SEM) in seconds for each treatment group were: saline = 11.0 ± 1.5; D-Cystine diME = 12.9 ± 1.0; Morphine = 16.3 ± 1.8; Morphine + D-Cystine diME = 16.9 ± 1.0. Using a repeated measures ANOVA and Tukey-Kramer (P < 0.05 for significance and differences between and across trials) there was no significant difference across trials as well as no significant interaction between the trials and the drugs (P > 0.05 for both). There was a significant drug effect (P < 0.05). There was a significant difference between morphine and saline (P < 0.001) as well as Morphine + D-Cystine diME and saline (P < 0.01). There was not a significant difference between D-Cystine diME and saline (P > 0.05). D-Cystine diME was significantly different from morphine (P < 0.01), and significantly different from morphine + D-Cystine diME (P < 0.05). Morphine and morphine + D-cystine diME was not different from each other (P > 0.05). See summary results below.

**Supplemental Figure 6.** Paw-withdrawal latencies (Hargreaves test) in rats pretreated with saline (n = 3 rats), D-cystine diME (500 µmol/kg, IV; n = 3 rats), morphine sulfate (1 mg/kg, IV, n = 3 rats) and a combination of morphine + D-cystine diME (n = 3 rats). The data are presented as mean ± SEM. *P < 0.05, morphine alone *versus* saline or morphine plus D-cystine diME *versus* L-cystine diME alone

**Von Frey Test – see Supplemental Figure 7.** Using the same test groups, 8 rats for saline, 3 rats for D-cystine diME, 3 rats for morphine + D-cystine diME, and 10 for Morphine alone, we found that force (in grams) needed to initiate hindpaw withdrawal was (mean ± SEM): Saline = 139 ± 7; D-cystine diME = 144 ± 8; Morphine = 177 ± 8; Morphine + D-cystine diME = 176 ± 5. Saline D-cystine diME values were similar to one another (P > 0.05). Morphine alone and morphine + D-cystine diME were significantly different from saline or D-cystine diME alone (P < 0.001 for both comparisons). The morphine values and morphine + D-cystine diME values were similar to one another (P > 0.05). The summary figure is below.

**Supplemental Figure 7.** Paw-withdrawal latencies (Von Frey test) in rats pretreated with saline (n = 8 rats), D-cystine diME (500 µmol/kg, IV; n = 3 rats), morphine (1 mg/kg, IV, n = 10 rats) and a combination of morphine + D-cystine diME (n = 3 rats). Data are presented as mean ± SEM. *P < 0.05, morphine alone *versus* saline or morphine + D-cystine diME *versus* L-cystine diME alone.

**References**

S1. Hargreaves, K., Dubner, R., Brown, F., Flores, C. & Joris, J. A new and sensitive method for measuring thermal nociception in cutaneous hyperalgesia. *Pain* **32**, 77-88 (1988).

S2. Carstens, E. & Wilson, C. Rat tail flick reflex: magnitude measurement of stimulus-response function, suppression by morphine and habituation. *J. Neurophysiol*. **70**, 630-639 (1993).

S3. Chesler, E.J., Wilson, S.G., Lariviere, W.R., Rodriguez-Zas, S.L. & Mogil, J.S. Identification and ranking of genetic and laboratory environment factors influencing a behavioral trait, thermal nociception, via computational analysis of a large data archive. *Neurosci. Biobehav. Rev*. **26**, 907-923 (2002).

S4. Le Bars, D., Gozariu, M. & Cadden, S.W. Animal models of nociception. *Pharmacol. Rev*. **53**, 597-652 (2001).

S5. Mogil, J.S., Chesler, E.J., Wilson, S.G., Juraska, J.M. & Sternberg, W.F. Sex differences in thermal nociception and morphine antinociception in rodents depend on genotype. *Neurosci. Biobehav. Rev*. **24**, 375-389 (2000).

S6. Mogil, J.S. & McCarson, K.E. Identifying pain genes: bottom-up and top-down approaches. *J. Pain* **1**(3 Suppl), 66-80 (2000).

S7. Kocevski, D. & Tvrdeić, A. The effect of repeated daily measurements on paw withdrawal latencies in Hargreaves test. *Collegium Antropologicum*. **32**(Suppl 1), 93-97 (2008).

S8. Hashimoto, M., Hossain, S., Shimada, T., Sugioka, K., Yamasaki, H., Fujii, Y., Ishibashi, Y., Oka, J-I. & Shido, O. Docosahexaenoic acid provides protection from impairment of learning ability in Alzheimer’s disease model rats. *J. Neurochem*. **81**, 1084-1091 (2002).

S9. Ikemoto, A., Ohishi, M., Sato, Y., Hata, N., Misawa, Y., Fujii, Y., Okuyama, H. Reversibility of n-3 fatty acid deficiency-induced alterations of learning behavior in the rat: level of n-6 fatty acids as another critical factor. *J. Lipid Res*. **42**, 1655-1663 (2001).

S10. McNamara, R.K., Sullivan. J. & Richtand, N.M. Omega-3 fatty acid deficiency augments amphetamine-induced behavioral sensitization in adult mice: prevention by chronic lithium treatment. *J. Psychiatric. Res*. **42**, 458-468 (2008).
